# Neighborhood Cohesion, Acculturation, and Oral Health Problems Among Older Chinese American Immigrants

**DOI:** 10.1093/geroni/igaa057.2988

**Published:** 2020-12-16

**Authors:** Weiyu Mao, Bei Wu, Iris Chi, Wei Yang, XinQi Dong

**Affiliations:** 1 University of Nevada, Reno, Reno, Nevada, United States; 2 New York University, New York, New York, United States; 3 University of Southern California, Los Angeles, California, United States; 4 Rutgers University, New Brunswick, New Jersey, United States

## Abstract

The influences of neighborhood characteristics remain understudied in relation to oral health, especially within the context of immigration. Acculturation exerts influences on the oral health of immigrants. This study investigated the relationship between neighborhood cohesion and oral health problems among older Chinese American immigrants and examined the moderating role of acculturation in such a relationship. The working sample included 3,157 older Chinese American immigrants aged 60 years or older from the baseline of the Population Study of Chinese Elderly in Chicago. Stepwise logistic regression models with interaction terms were conducted. Individuals experiencing higher levels of neighborhood cohesion reported a lower likelihood of having oral health problems. The protective effect of neighborhood cohesion against having oral health problems was stronger when individuals resided in ethnic enclaves such as Chinatown. To promote optimal oral health, interventions need to account for individuals’ perceptions and levels of integration into their neighborhoods and communities.

